# Precise in situ etch depth control of multilayered III−V semiconductor samples with reflectance anisotropy spectroscopy (RAS) equipment

**DOI:** 10.3762/bjnano.7.171

**Published:** 2016-11-21

**Authors:** Ann-Kathrin Kleinschmidt, Lars Barzen, Johannes Strassner, Christoph Doering, Henning Fouckhardt, Wolfgang Bock, Michael Wahl, Michael Kopnarski

**Affiliations:** 1 Research Group Integrated Optoelectronics and Microoptics (IOE), Physics Department, University of Kaiserslautern, PO Box 3049, D-67653 Kaiserslautern, Germany; 2 Institut für Oberflächen- und Schichtanalytik (IFOS) GmbH, Trippstadter Str. 120, D-67663 Kaiserslautern, Germany

**Keywords:** broad area semiconductor lasers (BAL), dry-etch monitoring (RIE), precise etch depth control, reflectance anisotropy spectroscopy (RAS), III–V semiconductors

## Abstract

Reflectance anisotropy spectroscopy (RAS) equipment is applied to monitor dry-etch processes (here specifically reactive ion etching (RIE)) of monocrystalline multilayered III–V semiconductors in situ. The related accuracy of etch depth control is better than 16 nm. Comparison with results of secondary ion mass spectrometry (SIMS) reveals a deviation of only about 4 nm in optimal cases. To illustrate the applicability of the reported method in every day settings for the first time the highly etch depth sensitive lithographic process to form a film lens on the waveguide ridge of a broad area laser (BAL) is presented. This example elucidates the benefits of the method in semiconductor device fabrication and also suggests how to fulfill design requirements for the sample in order to make RAS control possible.

## Introduction

Reflectance anisotropy/difference spectroscopy (RAS/RDS) [1–5] is an established powerful method to monitor the epitaxial growth of monocrystalline semiconductor layers in situ [6–7] – for instance for molecular beam epitaxy (MBE). The RAS technique employs reflectometric as well as interferometric information, based on the difference in optical surface reflectivity for two orthogonally linearly polarized light waves with (nearly) normal incidence onto the sample surface.

In [8–9] we have investigated the applicability of the RAS method and equipment to monitor the current etch depth in situ in dry-etch processes with high accuracy and reproducibility. Dry-etching is an essential step of many lithographic processes, e.g., in semiconductor device fabrication, and offers several advantages over wet-etching as for instance the opportunity to achieve anisotropic etching and vertical etch flanks [10–11].

As the requirements for accuracy in microfabrication increase with decreasing structure size, in situ monitoring is gaining importance. There are various approaches to monitor and control the etch depth during dry-etching: With methods based on gas-phase analysis such as mass spectrometry of the residual gas [12] or optical gas-phase analysis such as optical emission spectroscopy [13–14] it is possible to achieve an end-point control [11,13,15]. In this case information on the actual etch depth can only be retrieved in connection with changes in material composition at the interfaces between any two layers. Other techniques are based on surface analysis. Those are mostly optical, for instance (laser) interferometry and reflectometry [16–17] or (spectroscopic) ellipsometry [18]. Also, combinations of several techniques are applied [19–21].

RAS incorporates some advantages. Usually for RAS reflectometric information is recorded for a broad spectral range of photon energies between approx. 1.5 and 5.0 eV. Since layered samples optically represent coupled Fabry–Perot resonators, interferometric information can also be extracted from parts of the spectrum (for not too small wavelengths). Fabry–Perot oscillations can be observed with changing layer thickness (the latter either increasing during an epitaxial process or diminishing during dry-etching). The current thickness of the etched layer can be determined in situ from single photon energy transients of just the average reflected intensity and, hence, any required etch depth can be achieved. Due to the broad spectral range even structures with many layers of different material composition may be monitored during a single etch process. Furthermore, RAS data contain information on the sample surface (etch front) morphology [9], which are indicative of the roughness.

In this contribution the high accuracy in etch depth control with RAS, which until now had been estimated only in our earlier publications [8–9], is demonstrated. Different etch depths have been monitored during etching of layered samples and the results are compared to secondary ion mass spectrometry (SIMS) measurements of the remaining layer thicknesses. Thus the accuracy stated earlier can be verified experimentally, for the first time. The benefit of dry-etch control with RAS is demonstrated for a realistic semiconductor device fabrication process, which is highly etch depth sensitive: i.e., the fabrication of a film waveguide lens on top of a broad area semiconductor laser ridge. The film lens is intended to guarantee laser operation in the fundamental transverse mode. An undesired deviation of the film lens layer thickness of only a few tens of nanometers may change the effective refractive index of the lens for the fundamental laser mode and hence the focal length of the lens, to which the lasers’ length is adapted, drastically. This fabrication procedure serves as an example for all processes, where no additional etch stop layer can be incorporated. The example also deals with the wafer layout necessary for RAS dry-etch monitoring.

## Results and Discussion

### Experimental details

Most of the III–V semiconductors under normal conditions feature optically isotropic bulk material due to their cubic crystal structure and consequently should not generate a RAS signal under normal incidence [1]. Nevertheless, optical anisotropies of the surface, originating, e.g., from specific surface reconstructions [1,5], give rise to a RAS signal. The surface sensitive RAS signal carries information about the current growth or etch front, respectively. As mentioned above, to monitor a monocrystalline surface with RAS, it is illuminated with perpendicularly incident linearly polarized light of a wide spectral range. Optical anisotropies of the sample surface (as mentioned above) break the symmetry and make the notion of two orthogonally linearly polarized light beams meaningful – even for surface-normal incidence. This leads to an elliptical polarization of the reflected light [5]. The reflected intensity is analyzed with respect to the orthogonal planes of polarization. Each of these planes incorporates a principal crystal axis. The RAS signal is defined as follows (written for a specific example of the relevant crystal axes):

[1]
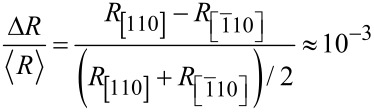


with the reflectances/reflectivities *R* of the relevant directions of linear polarization designated by Miller’s indices of the corresponding principal axes (in this example [110] and 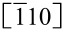) and the total average reflectance/reflectivity 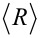 [3–4,6,8].

**Figure 1:**
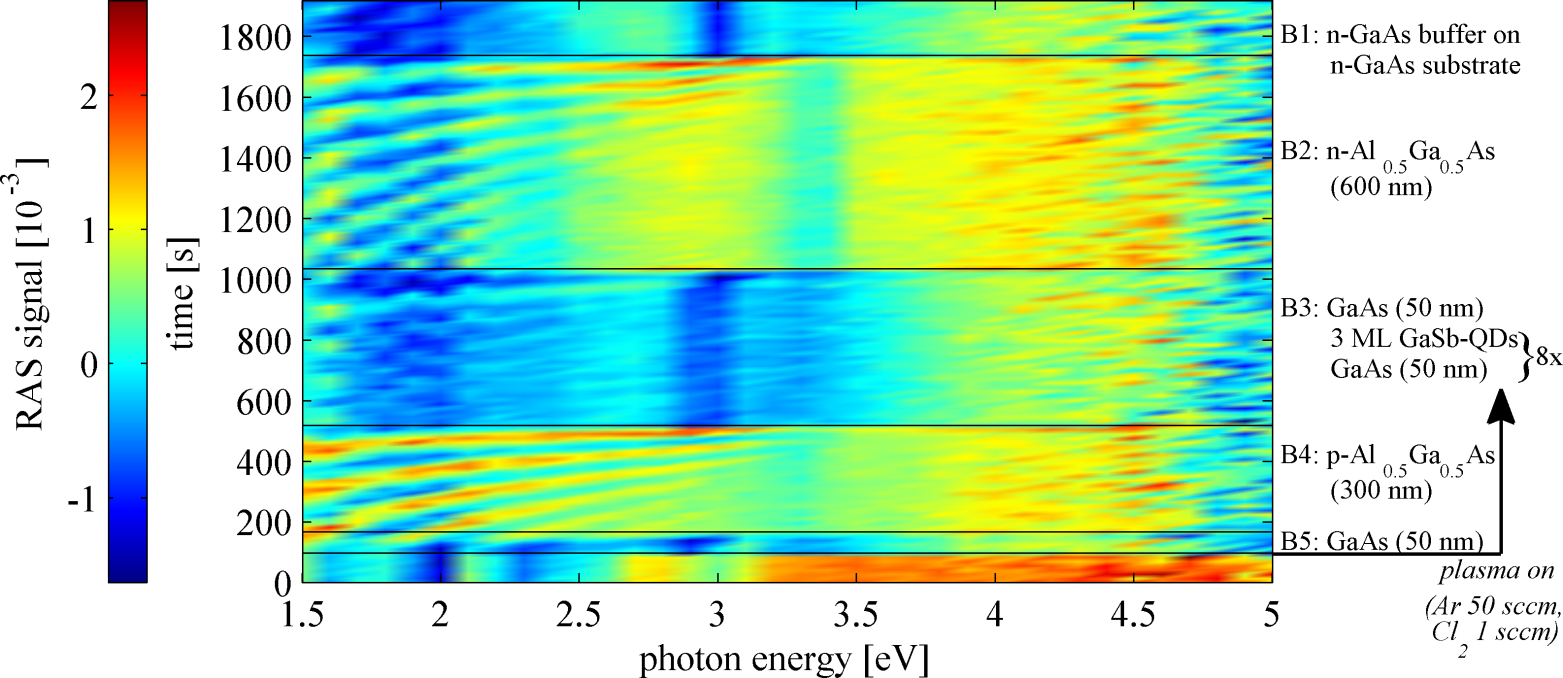
Color plot of the RAS signal during reactive ion etching (RIE) of a partially masked laser substrate (etch step I, see section “Application”). After turn-on of the plasma the different semiconductor layers of the laser material B5–B1 are etched (from bottom to top of the plot) (sample type B).

In case the signal is given as a function of photon energy, the term RAS spectrum will be used. Due to the rotation of the substrates during epitaxy the software of the epitaxy-RAS instrumentation typically ignores the sign of the RAS signal. The time evolution of the spectrum of this RAS signal is called a false-color plot or short color plot (see also [22]), since now the signal height is displayed color-coded (red for high, blue for low values, see Figure 1), while time is given on the ordinate. The color plot gives information on the state and evolution of the growth/etch front for the different points in time.

For dry-etching (RIE) the situation is slightly different and the (false-) color plots are based on the RIE-RAS signal according to Equation 1, which can be negative. Thus the range of colors for signal height coding covers the whole range from negative to positive values.

The multilayered samples under investigation in this contribution have been grown with molecular beam epitaxy (MBE) in a R450 MBE system from DCA Instruments Oy, Turku, Finland. Base and working pressure of the system are (8–9) × 10^−10^ hPa and (1–2) × 10^−10^ hPa, respectively.

**Figure 2:**
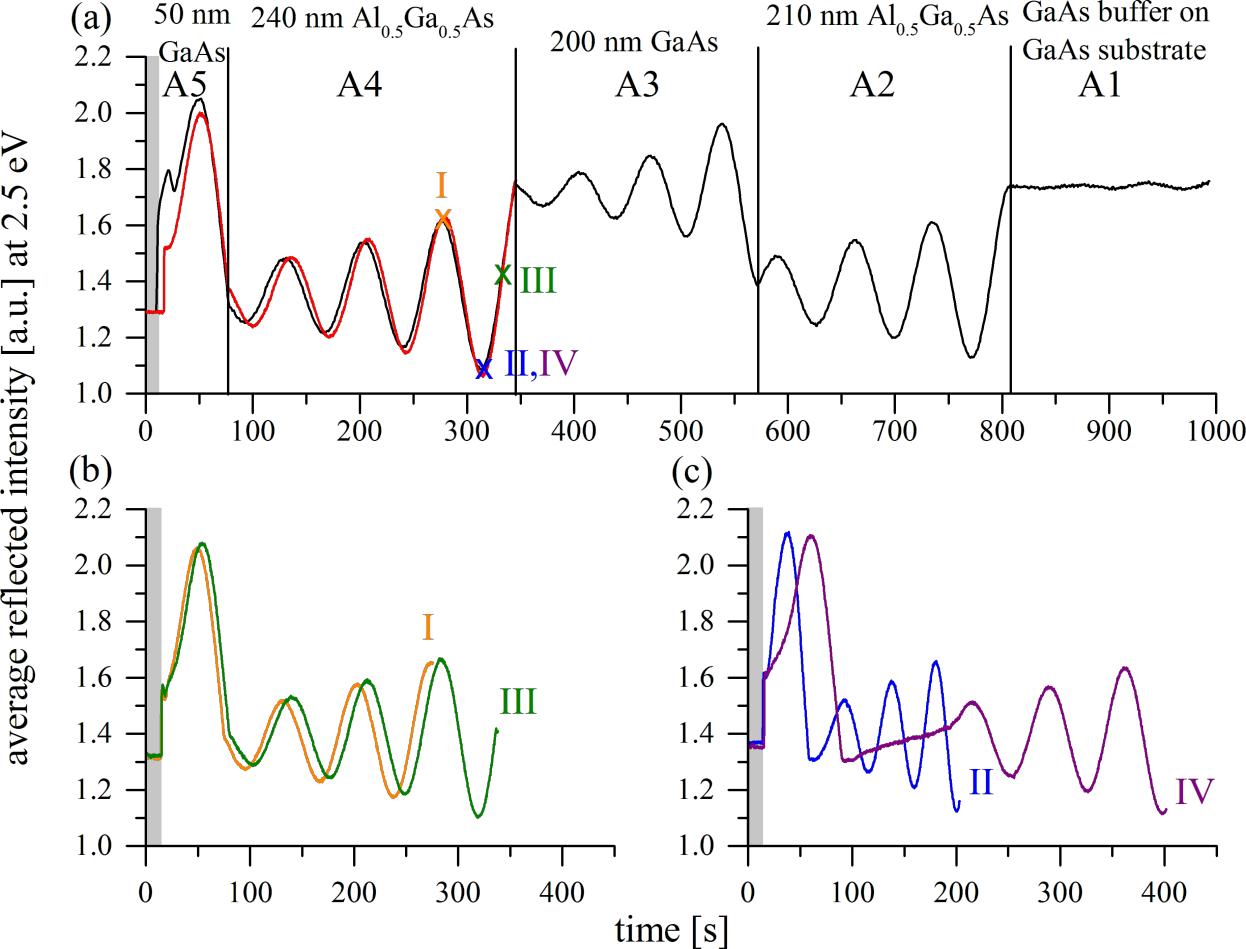
Transients of the average reflected intensity in arbitrary units (a.u.) at a photon energy of 2.5 eV during reactive ion etching of pieces of the sample of type A. a) Transient of a reference piece, which has been etched through all the MBE-grown semiconductor layers (black line) and of a piece etched through the upper two layers A4 and A5 only (red line). b) and c) Transients of the average reflected intensity for four pieces, where the etch process is stopped at three different points of the oscillating intensity (also marked in (a) as I, II, III, IV).

For the experiments concerning the accuracy of the etch depth determination, described in section “Experimental results”, a sample is comprised of several undoped GaAs and Al_0.5_Ga_0.5_As layers with different thicknesses, grown on a GaAs substrate (called sample type A, for details see Figure 2).

For the exemplarily mentioned application, described in section “Application”, a layer sequence of a semiconductor laser, mainly consisting of several layers of Ga(As)Sb quantum dots (QD) (as active material) embedded in GaAs, surrounded by n- and p-doped layers of Al_0.5_Ga_0.5_As (for the diode and waveguide structure), has been grown on an n-doped GaAs substrate (called sample type B, for details see Figure 1).

The samples are etched in a parallel plate reactor RIE system (MicroSys 350 from Roth & Rau, Wuestenbrand, Germany) using a bias voltage of 700 V. The plasma gas consists of Cl_2_ (1 sccm) and Ar (50 sccm), while the sample holder is backside cooled with He gas (10 sccm). Base pressure of the etching system is about (1–5) × 10^−6^ hPa, while the pressure during the etch process is about (1–2) × 10^−2^ hPa.

Chlorine based plasmas are commonly used for dry-etch processes of III–V semiconductors [23–28]. In our case 2 vol % of chlorine (1 sccm in 50 sccm argon) suffice to prevent the accumulation of debris from, e.g., the chamber walls and re-deposition of etched material on the sample surface. Moreover, these 2% enlarge the etch rate by a factor of about 5 compared to the case with pure Ar. The chlorine share should not exceed 20% though, because otherwise the RAS spectra will not be clearly characteristic of the etch front. Details on the determination of the optimum gas parameters, especially for etching GaAs and Al_0.5_Ga_0.5_As, and on the influence of the plasma gas composition on RAS can be found in [9].

To monitor the same samples both during their epitaxial growth and reactive ion etching (RIE) two similar EpiRAS instruments by Laytec, Berlin, Germany, are employed. In MBE growth RAS is well established meanwhile [6–7] and optical access is provided easily. The use of a RAS system in combination with RIE – especially a parallel plate reactor as in our case – requires a modification of the RIE vacuum chamber, more precisely of the upper electrode to provide optical access to the sample. The upper electrode and the upper flange have to be equipped with an optical window. The RAS system has to be installed above the RIE chamber; details are given in [8].

### Experimental results

From RAS (false-) color plots information about the composition and quality of a semiconductor layer or surface can be extracted. Usually RAS is used to monitor growth processes.

However, as already stressed, not only the growth, but also the inverse process, i.e., dry-etching, can be monitored in situ and controlled with RAS [8–9]. Figure 1 exemplarily shows a RAS (false-) color plot of a piece of the sample of type B from a RIE dry-etch process. Immediately after plasma turn-on, the early RAS data, resulting from monitoring the oxidized GaAs layer [9] (lower part of Figure 1), change due to the start of the etching. During the process each semiconductor layer with its specific material composition can be identified by its characteristic region of the RAS color plot.

A comparison of color plots of growth and dry-etching reveals the similarity of the surface evolutions (one of them time-reversed due to erosion instead of growth) [8]. For a decreasing thickness of a layer of the sequence (a shrinking Fabry–Perot resonator thickness) there is a temporal evolution due to the current etch depth, which adds interferometric information, as stated earlier.

While first results on the similarity of the average reflected intensity of growth and etch processes have been investigated [8], similarities of the genuine RAS signal still have to be examined further. RAS monitoring of etch processes holds great potential for investigating surfaces during etching without the need for further techniques (as, e.g., reflection high-energy electron diffraction (RHEED)), which might not be applicable in some set-ups.

Recording a RAS color plot is time-consuming, i.e., monitoring a single RAS spectrum from 1.5–5.0 eV photon energy with a step size of 0.1 eV during reactive ion etching (the substrate does not rotate) takes about 15 s. However the plot itself contains more information than needed for etch depth control. Monitoring just the average reflectance/reflectivity 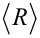 (the denominator in Equation 1) or even just the average reflected intensity at a single significant photon energy (or just a few) saves data collection time.

In the RAS signal, according to Equation 1, Fabry–Perot oscillations could be suppressed, if both reflectivities in the numerator changed very similarly. On the contrary, as can be seen, e.g., in Figure 2, the average reflected intensity, corresponding to the DC output signal of the detector at a photon energy of 2.5 eV (sample of type A), exhibits oscillations in time for the etched materials GaAs and Al_0.5_Ga_0.5_As. Those oscillations originate from the changing thickness of the layer currently being etched and can be used to determine the total residual thickness of the layer and to stop the etch process after reaching a certain etch depth. The etched thickness Δ*d* of a layer can be calculated according to:

[2]
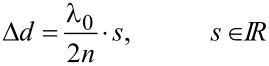


where λ_0_ is the (vacuum) wavelength of the light, *n* is the (average) refractive index of the investigated layer and *s* stands for the number of periods of the oscillation (which is a real number here).

Different layer compositions can be recognized via their offset values of the intensity [9]. The amplitude of the oscillations increases with decreasing layer thickness, presumably due to less absorption.

To check the accuracy of this technique, four pieces of about the same size (typically about an eighth of a 2-inch wafer) of a MBE-grown sample of type A are etched separately using the same etch parameters. The etch processes are stopped at three different points of the RAS intensity oscillation for the first Al_0.5_Ga_0.5_As layer A4 (I, II, III, IV) (Figure 2a). The stop points have been chosen to be right before reaching the interface between the layers Al_0.5_Ga_0.5_As (A4) and GaAs (A3). Figure 2b and c show the corresponding transients of the average reflected intensity. The etch processes of piece II and IV have been stopped at about the same point of oscillation. Striking is that during the etching of piece IV an etch delay for an unknown reason occurs (Figure 2c), as will be discussed below.

The reference values for the thicknesses of the grown semiconductor layers have been determined using scanning electron microscopy (SEM) with an SU8000 system by Hitachi. Thus a piece of a sample of type A had to be cleaved and the resulting facet had to be investigated with the SEM with a tolerance in the determination of the layer thicknesses of ±5% maximum.

**Figure 3:**
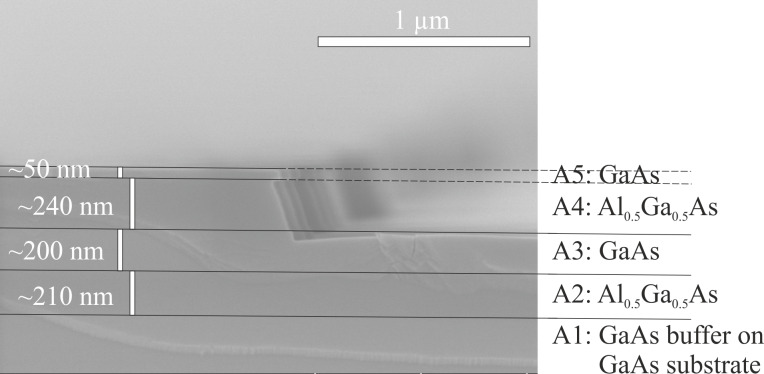
Scanning electron microscopy (SEM) image of a facet of a lithographically masked and then reactive-ion-etched piece of the sample of type A. The mask material (i.e., photo-resist) has been removed wet-chemically. The different semiconductor layers with various thicknesses can be recognized. The dry-etch process has been stopped after reaching the second GaAs layer (A3).

Figure 3 shows an SEM image of a cleaved facet of a lithographically masked and then reactive-ion-etched piece of the sample of type A. The etch process was stopped right after reaching the second GaAs layer (A3) (see red line in Figure 2a). The photoresist etch mask was removed before cleaving the sample. As can been seen RAS control of dry-etching enables the RIE operator to stop the etch process at a requested point with an accuracy of a few tens of nanometers or even a few nanometers (see Table 1).

Only at the typical unavoidable trench at the bottom of the flank the etching has proceeded slightly deeper [15]. However, the typical trench depth can be anticipated to achieve the desired etch depth – depending on the relevance of the trench depth for the device function.

To examine the reliability of RAS in controlling the etch depth, the etched sample pieces I–IV (corresponding to specific etch times – see Figure 2) and, for reference, a not reactive-ion-etched piece of the same sample (type A) are investigated by secondary ion mass spectrometry (SIMS), using a TOF–SIMS IV instrument from ION-TOF GmbH, Muenster, Germany. Applying the so-called dual beam depth profiling technique with 2 keV Cs^+^ as sputter ions and 20 keV Bi^3+^ as analysis ions, the thickness of the sputtered Al_0.5_Ga_0.5_As (i.e., Al-containing) layer can be determined indirectly by examining the secondary ion intensity of the resulting CsAl molecule over sputter time. The specific sputter time, which is needed to remove the complete Al_0.5_Ga_0.5_As layer A4 of the unetched reference sample *t*_ref_ corresponds to a layer thickness of *d*_ref_ = 240 nm, known from SEM inspection (related sputter time *t*_ref_ = 224 s). This value is taken as a reference in determination of the remaining thicknesses *d*_a_ (remaining after etching and sputtered during the SIMS process) of the sample pieces I, II, III, and IV, assuming an identical sputter rate for the used SIMS analysis conditions in all cases:

[3]
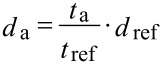


**Figure 4:**
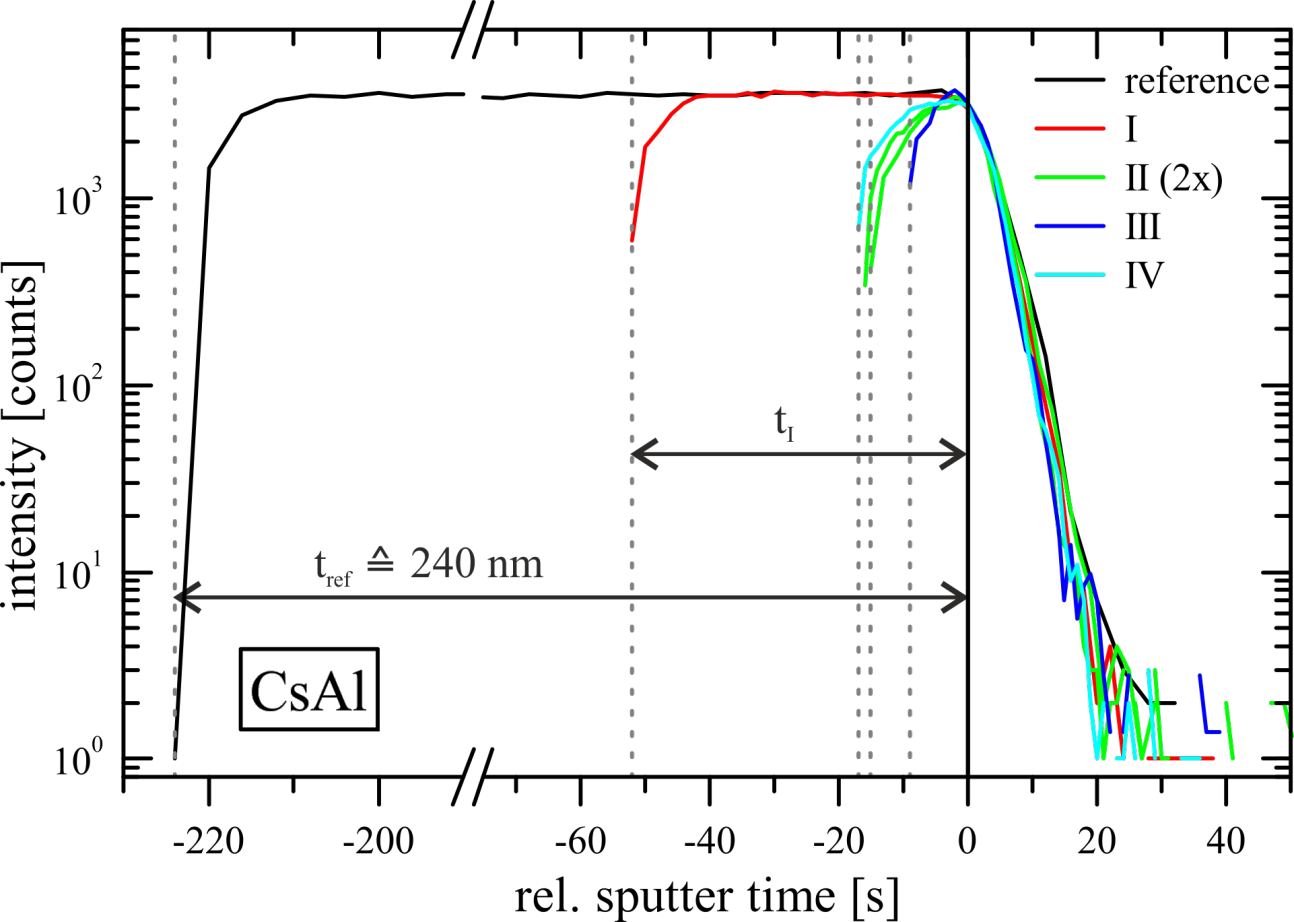
SIMS intensity – sputter time profile of the CsAl-signal with logarithmic scaling. Displayed are profiles for the sample pieces I–IV and for the reference sample piece of the same type. The sputter time on the abscissa is shifted for simplicity so that the interface between the Al_0.5_Ga_0.5_As and the GaAs layer (indicated by a steep decrease of the CsAl signal) is located at the time *t* = 0 for all analyzed sample pieces. The relevant sputter times *t*_ref_ for the reference and *t*_I_ for sample piece I have been marked exemplarily.

Figure 4 shows the detected CsAl intensity with logarithmic scaling as a function of sputter time, which is shifted for simplicity such that the interface between the Al_0.5_Ga_0.5_As layer and the GaAs layer (indicated by the steep decrease of the CsAl signal) is located at the time *t* = 0 for all analyzed samples.

Table 1 gives an overview of the remaining Al_0.5_Ga_0.5_As A4 layer thicknesses of sample pieces I–IV calculated with the numbers *s* of periods of the Fabry–Perot oscillations according to Equation 2. For the evaluation the refractive index of layer A4 is adjusted to be *n* = 3.79 for a photon energy of 2.5 eV, according to the real-valued number *s* of measured periods of the Fabry–Perot oscillation for the known layer thickness of 240 nm. The reference sample shows 3.67 periods of oscillation of the average reflected intensity for the Al_0.5_Ga_0.5_As layer A4. The number *s* has subsequently been determined from the average period and the total etch duration of each layer. This is particularly recommended for regular etch processes, due to its high accuracy. For irregular processes, for example processes with some delay (see Figure 2c, IV, showing an etch delay for an unknown reason), this approach cannot be employed. In this case the actual start and end phases of the oscillation have to be estimated directly, which can be less accurate. On the other hand in this case, no matter how large the delay is, the number 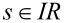 of periods of the oscillation and hence the etch depth can be retrieved. This forms another strong advantage of the RIE etch depth control via RAS.

**Table 1:** Remaining Al_0.5_Ga_0.5_As A4 layer thicknesses for the differently etched sample pieces and the reference (see Figure 2). The layer thickness has been determined according to Equation 2 by using the numbers *s* of periods of the oscillations at a photon energy of 2.5 eV. The calculated results are compared to results received by secondary ion mass spectrometry (SIMS) in combination with SEM inspection.

sample	number of periods of the oscillation *s*	(etched) layer thickness according to Fabry–Perot (nm)	remaining layer thickness RAS (nm)	sputter time SIMS Cs^+^ *t*_a_ (a = ref,I,II,III,IV) (s)	remaining layer thickness *d*_a_ SIMS (nm)

reference (piece 1)	3.67	240	**0**	–	**–**
reference (piece 2)	–	–	**–**	224	**0**
I	2.78	182.2	**57.8**	52	**55.7**
II	3.36	220.1	**19.9**	15	**16.1**
III	3.54	231.7	**8.3**	9	**9.7**
IV	3.05	197.7	**40.3**	17	**18.2**

Sample piece I has the thickest remaining Al_0.5_Ga_0.5_As A4 layer of all etched pieces, as expected. The remaining layer thickness is 57.8 nm (24.1% of the initially MBE-grown 240 nm). Sample piece II has 19.9 nm (8.3%) of A4 layer left and piece III 8.3 nm (3.5%).

A comparison of these results with the calculated SIMS thickness values shows a maximum absolute deviation of ≈4 nm (except for the case of sample piece IV with an etch delay, already mentioned). Due to this good agreement of the results of the two completely different analysis methods, this is a strong indication that the RAS method delivers a reliable value of the etched layer thickness.

The in situ accuracy of etch-depth control by RAS mainly depends on the uncertainty in determination of the exact phase of the Fabry–Perot oscillation and on the time needed to stop the etch process, i.e., to switch off the plasma [9]. According to a medium-case estimate a quarter of an oscillation period can be identified clearly, which corresponds to a change of the layer thickness of Δ*d* = λ/8 [8–9]. In the current example this means: 2.5 eV (photon energy) ↔ λ_0_ ≈ 496 nm (wavelength in vacuum) ↔ λ ≈ 131 nm (wavelength in the semiconductor) 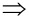 Δ*d* = λ/8 ≈ 16 nm. Thus a resolution of the etch depth of <16 nm should be achievable.

### Application

In situ monitoring of a semiconductor surface during dry-etching is an important improvement for microtechnology. With the method described above, the etch depths, even of multilayered samples, can be determined in situ during the etch step. Thus etch parameters can be adjusted dynamically. Generally, a higher precision in etch depth can be achieved and the process yield can be increased.

For some applications the accuracy of a certain film thickness is essential and even a few nanometers may be decisive. Thus the dry-etch process does not only have to be monitored, but also the etch depth needs to be controlled precisely in real time.

**Figure 5:**
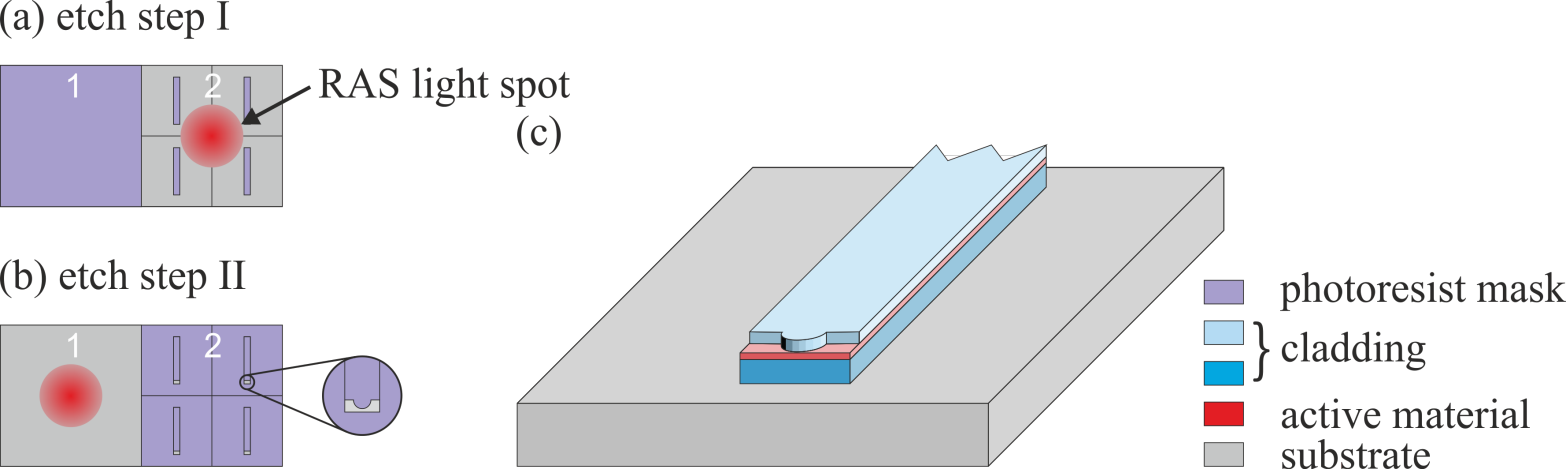
Illustrations of the wafer layout and sample design (required in the example) to monitor the etch processes for structured laser ridges. Parts (a) and (b) show a top view of the sample during the two required etch steps: Etch step I: The control window is covered (area 1), the remaining part of the sample is patterned (area 2). The RAS light spot (red circle) is focused on the patterned area. Etch step II: The control window is not masked (area 1), the laser ridges are covered, except for the lens area (area 2). c) Resulting single structured ridge laser without any mask drawn in perspective.

One particular application is presented here – see Figure 5. With this example a way to monitor several etch steps of a sample is shown and a possibility to incorporate sample design features required for RAS control is given.

A common type of semiconductor laser is a ridge waveguide laser [29] – with a broad active region and ridge in our case. Broad area lasers (BAL) have the advantage of high output powers. Nevertheless, a serious drawback is the multi-transverse-mode operation [30–34]. To achieve operation in the fundamental transverse mode only, the higher order modes have to be suppressed. This can be realized by monolithically integrating a transverse mode selector into a BAL [35]. In our current attempt (among other features) a film waveguide lens [36] on the laser ridge is employed for this reason.

The focal length of the film waveguide lens for the fundamental transverse mode strongly depends on the difference of the effective refractive indices Δ*n* = *n*_1_ – *n*_0_ of the lens area (*n*_1_) and the area beyond the lens (*n*_0_, here the unmodified laser ridge). In our case, to define the waveguide lens, just the upper cladding is supposed to be etched away. For the current configuration of the laser, unintentional etching of just 50 nm into the active layer may cause an undesired deviation of the effective refractive index of up to Δ*n*_1_ = 0.0146. This corresponds to a deviation of the focal length of about 1400 nm (about 50% of the original focal length). Since attaining the right focal length is of extreme importance for transverse mode selection in our case, precise etch depth control is essential.

To fabricate the described laser, a semiconductor layer sequence (sample of type B) is masked with the desired stripe pattern and the unmasked regions are deeply etched with reactive ion etching (lithographic process I, see Figure 5a). Here a soft mask of photoresist has been used to define the stripes (laser ridges). Adjacent to each of the ridges the upper cladding, the active region, and the lower cladding are removed by dry-etching to achieve strong optical confinement (compare [35]).

In a second lithographic process (process II, see Figure 5b) and etch step the laser ridge itself is being structured to alter the effective refractive index locally and thus to achieve a film waveguide lens in part of the ridge. Therefore, again a photoresist mask is used to define the desired pattern on the laser ridge in combination with RIE. (The shape of the etched area has to be concave to achieve the effect of a converging film waveguide lens for the guided light (Figure 5c).)

To monitor the etch process of a masked sample a small unmasked area/window has to be provided for analyses, which is a common technique in semiconductor technology, while different measurement techniques might be the reason for the sacrificial window. In our case the window is needed for the RAS light spot, which has a diameter of about 4 mm, and should have a size of about (4 × 4) mm^2^.

In etch step I, i.e., the definition of the laser ridge, the RAS light spot is focused on the masked area of the sample (Figure 5a). This is not a problem, since the ridges account for only about 5% of the illuminated surface area. The control window is also covered with the photoresist to keep it even with the height of the laser ridge. This is important to be able to monitor the process during etch step II (Figure 5b). The first photoresist mask is substituted by a second mask, which only partially covers the laser ridges in order to structure the ridges themselves. Now the control window is not covered and can be used to monitor the etch process with RAS.

**Figure 6:**
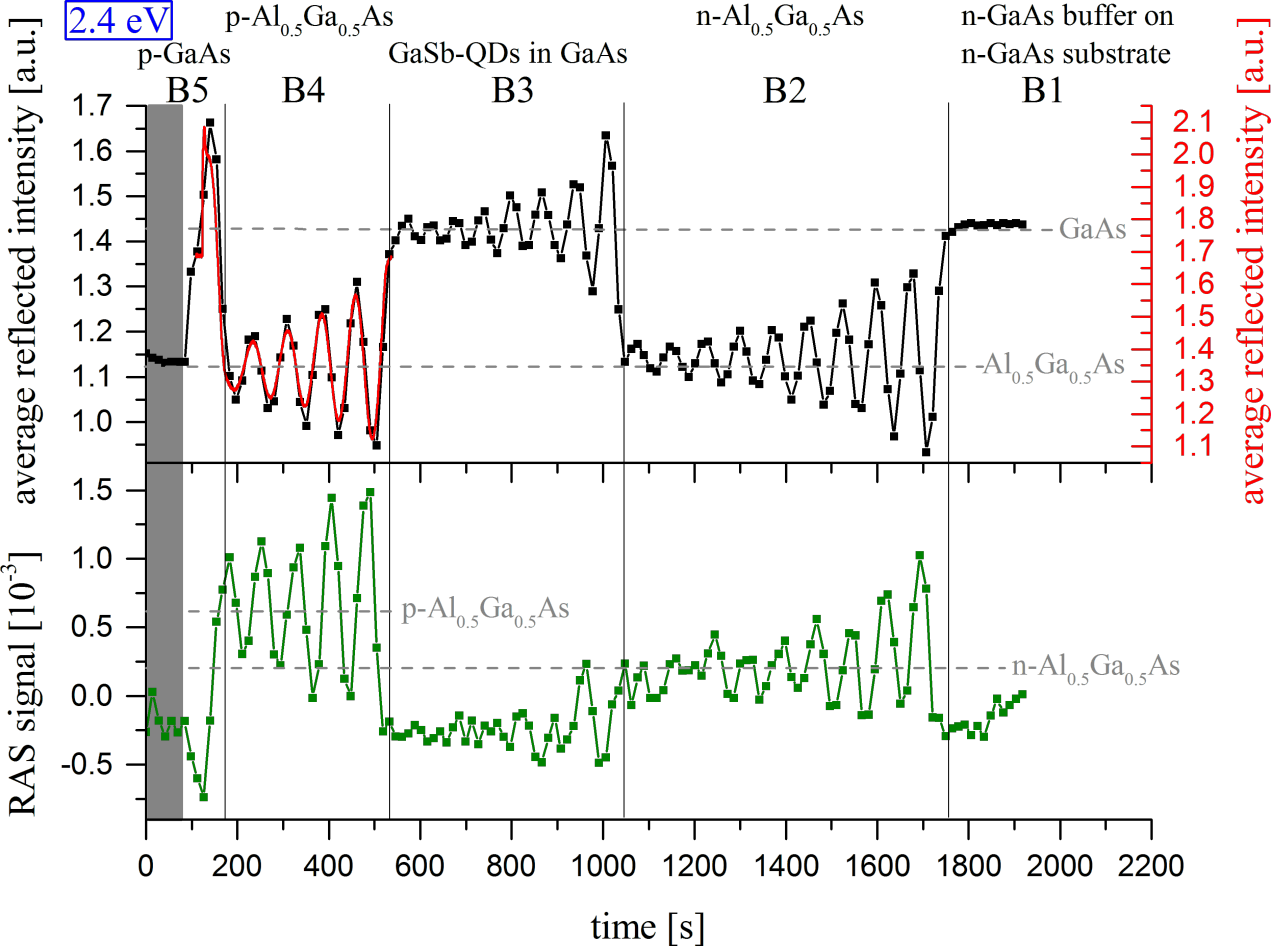
Single transients of the average reflected intensity (top) and the RAS signal (bottom) at 2.4 eV of the etch process to define the laser ridges (etch step I; dots interpolated with lines, black and green) and a single transient of the average reflected intensity (top) of the etch process to define the film lenses (etch step II, solid red line). In step II the etch process has been stopped after removing both the p-GaAs capping layer (B5) and the upper p-Al_0.5_Ga_0.5_As cladding (B4).

Figure 1 (far above) shows the RAS color plot of etch step I, the definition of the laser ridges/stripes. As soon as the plasma is switched on, marked on the right in Figure 1, the different layers of the laser material are etched – starting with the highly doped p-GaAs capping layer (B5), then the upper p-Al_0.5_Ga_0.5_As cladding (B4), the active area with eight layers of Ga(As)Sb quantum dots (QD) between 50 nm thick GaAs barriers (B3), the lower n-Al_0.5_Ga_0.5_As cladding (B2) and, finally, the n-GaAs buffer and substrate (B1). The different properties of the layers during etching become particularly obvious, when taking a look at the single transients of the average reflected intensity at a specific photon energy. A photon energy of 2.4 eV is chosen, because Fabry–Perot oscillations of the RAS signal for the individual semiconductor layers can be clearly observed here (Figure 6, top).

As marked in the plot, the offset level of the average reflected intensity for the oscillations varies for the individual materials, i.e., GaAs and Al_0.5_Ga_0.5_As (Figure 6, top). A closer look at the transients reveals another important advantage of dry-etch monitoring with RAS: The RAS signal provides for information on the doping of the materials; p-doped and n-doped Al_0.5_Ga_0.5_As do have different offset/mean levels of the oscillating signal (Figure 6, bottom).

The main reason to monitor the uncritical etch step I (apart from being able to react to irregularities in the etch process) is to determine the desired point, where the process has to be stopped in etch step II, from the recorded data. Etch step II is stopped after removing both the capping layer (B5) and the upper cladding layer (B4) and right before the active region of the lasers (B3) is reached.

In etch step II the etch process in the control window is monitored, which provides information about the progress of the etching of the uncovered lens area on the laser ridge.

**Figure 7:**
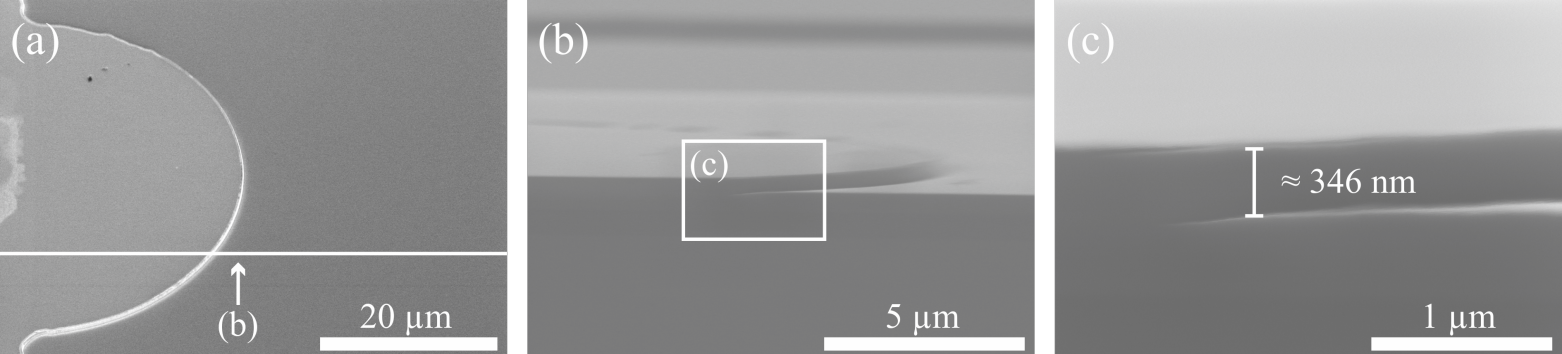
Scanning electron microscope (SEM) images of a film waveguide lens on a laser ridge. (a) Tilted top-view of the film lens. The lighter gray part on the left is the elevated part of the laser ridge compared to the lens area. (b) Profile of the film lens step of the laser cleaved alongside (compare line in (a)), (c) Zoomed-into profile of the film waveguide lens with an average height of 346 nm.

Figure 7 shows scanning electron microscope (SEM) images (micrographs) of a film waveguide lens on a laser ridge. The average height of the etched step is about (346 ± 17) nm (tolerance due to the SEM measurement), deviating from the desired 350 nm by about 4 nm only. (In our specific case the depth of the unavoidable trench has to be taken into account, since it changes the effective refractive index of the guided modes. However, it can be considered before etching, because typical trench depths are well-known for different etch parameters.) Thus with this highly etch-depth sensitive process a real benefit of on-line control is illustrated.

## Conclusion

Reflectance anisotropy spectroscopy (RAS) can be used to monitor monocrystalline III–V semiconductor sample surfaces in situ during reactive ion etching (RIE). Using the recorded reflectometric and interferometric data, represented for example by the average reflected intensity over time, in situ etch depth control of multilayered samples can be performed with accuracies better than 16 nm. Comparative SIMS results show a deviation from the etch depths evaluated from the Fabry–Perot oscillations of only about 4 nm in optimal cases.

For highly etch depth sensitive processes controlling the etch depth of the sample in situ is essential. Such a process is presented with the integration of a film waveguide lens on the waveguide ridge of a broad area semiconductor laser. This example of semiconductor device fabrication demonstrates the high accuracy and the benefits of the described method. It is directly applicable to samples with small photoresist-masked areas. Otherwise, a control window of about (4 × 4) mm^2^ for the RAS light spot on the sample has to be provided, a technique, which is not uncommon in microtechnology.

The described method to terminate an etch process at a desired etch depth has the potential to become an important tool in semiconductor device fabrication. Monitoring etch processes with RAS equipment does not only offer the possibility to analyze interferometric data and, hence, etch depth or rate, but also to gain information on other material properties of interest.

Information on surface morphology of a monocrystalline semiconductor sample can be extracted – either on an atomic scale due to specific surface reconstructions [3,5,37–38], known from various studies on epitaxial growth, or on surface roughness due to ion bombardment [9,39–41].

Also, information on the composition of compound semiconductors [1] as well as the doping of the etched layers (related to the offset/mean value of the Fabry–Perot oscillations) are included [9,42–44].

In the future, RAS equipment might even offer the possibility to monitor the etch depth with an accuracy down to a single monolayer, as might be concluded from short-period oscillatory RAS signals during semiconductor growth [38,45–47].
